# Incentivizing for Truth Discovery in Edge-assisted Large-scale Mobile Crowdsensing

**DOI:** 10.3390/s20030805

**Published:** 2020-02-02

**Authors:** Jia Xu, Shangshu Yang, Weifeng Lu, Lijie Xu, Dejun Yang

**Affiliations:** 1 Jiangsu Key Laboratory of Big Data Security and Intelligent Processing, Nanjing University of Posts and Telecommunications, Nanjing 210023, China; 1017041230@njupt.edu.cn (S.Y.); luwf@njupt.edu.cn (W.L.); ljxu@njupt.edu.cn (L.X.); 2 Department of Computer Science, Colorado School of Mines, Golden, CO 80401, USA; djyang@mines.edu

**Keywords:** mobile crowdsensing, incentive mechanism, edge computing, truth discovery

## Abstract

The recent development of human-carried mobile devices has promoted the great development of mobile crowdsensing systems. Most existing mobile crowdsensing systems depend on the crowdsensing service of the deep cloud. With the increasing scale and complexity, there is a tendency to enhance mobile crowdsensing with the edge computing paradigm to reduce latency and computational complexity, and improve the expandability and security. In this paper, we propose an integrated solution to stimulate the strategic users to contribute more for truth discovery in the edge-assisted mobile crowdsensing. We design an incentive mechanism consisting of truth discovery stage and budget feasible reverse auction stage. In truth discovery stage, we estimate the truth for each task in both deep cloud and edge cloud. In budget feasible reverse auction stage, we design a greedy algorithm to select the winners to maximize the quality function under the budget constraint. Through extensive simulations, we demonstrate that the proposed mechanism is computationally efficient, individually rational, truthful, budget feasible and constant approximate. Moreover, the proposed mechanism shows great superiority in terms of estimation precision and expandability.

## 1. Introduction

Mobile crowdsensing is a human driven activity that leverages pervasiveness of wireless connectivity, various mobile devices with built-in sensing capabilities, and inherent user mobility to create dense and dynamic data sets, which can effectively characterize our environments. Mobile crowdsensing has become an efficient approach to data acquisition in large-scale sensing applications, such as photo selection [1], public bike trip selection [2] and indoor positioning systems [3].

Most existing mobile crowdsensing systems [4,5,6] depend on the cloud service to collect/aggregate sensing data, allocate tasks, estimate the truth and incentivize the mobile users. Cloud based mobile crowdsensing systems have some obvious drawbacks, e.g., weak expansibility for large-scale crowdsensing due to the computing and communication congestion on the cloud severs, difficulty of recognizing the fake locations of sensing data, high risk of data security and user privacy exposure.

Recently, edge computing based mobile crowdsensing architecture has been proposed [7,8,9,10,11] to solve above issues. The main benefits of edge computing based architecture for large-scale mobile crowdsensing are as follows:

- Reduce the computational complexity: the edge computing based mobile crowdsensing architecture can parallelize the computing through offloading the computing from the cloud to multiple edge servers.
- Decrease the latency: There is less or no necessary communication between the cloud and the mobile users.
- Location-awareness: Most mobile crowdsensing tasks are location dependent [12,13,14]. The edge computing resources (such as base stations and access points) are usually with specific locations. Since the edge servers only collect the sensing data within their deployment area, it is easy to verify the location property of sensing data. For example, the crowdsensing of noise monitoring or traffic monitoring for specific locations. The sensing data largely depends on the accuracy of location information.
- Flexible data processing. Edge computing based mobile crowdsensing brings the flexibility of local data processing (such as aggregation, truth discovery and inference of temperature, noise level, transportation and air condition for specific areas) in edge servers. For example, the edge cloud can be used to estimate the local noise level or analyze local traffic video, which do not need to be executed in the deep cloud.
- Reduce privacy threats: The sensing data is distributed in multiple edge servers. The distributed storage of sensing data in multiple edge servers not only enhances security of data but also reduces privacy threats of users. For example, the crowdsensing data of personal living environment/photos are private information, and are more suitable to be processed in edge servers.

There have already been some studies of mobile crowdsensing, which use edge computing to achieve some goals, e.g., edge computing based data processing [7,15], privacy-preserving [8,16,17], reputation management [9], task offloading of vehicular crowdsensing applications [10] and extraction of the environmental information [11]. However, there is no truth discovery and incentive mechanism design in the state of the art.

In this paper, we aim to develop an integrated solution to stimulate users to improve the quality of sensing data. There are two key problems that need to be solved:

- How to estimate the true value (truth discovery) under edge computing based mobile crowdsensing architecture?
- Further, how to incentivize the strategic users to contribute more for truth discovery?

**Figure 1:**
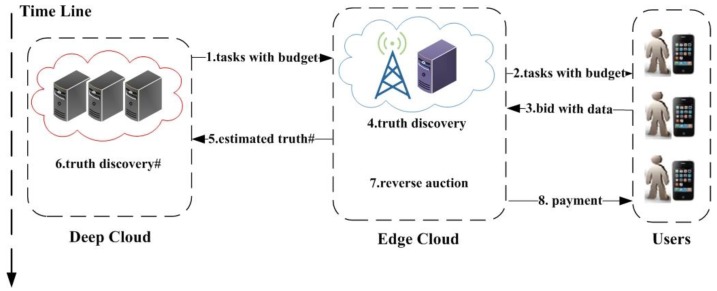
Edge-assisted large-scale mobile crowdsensing (# represents the optional operation).

We consider a mobile crowdsensing system consisting of a platform, a set of edge clouds and a set of users. The platform resides in the deep cloud. Each edge cloud is responsible for collecting sensing data in its coverage area. We model the crowdsensing process as a sealed reverse auction. First, the platform distributes the crowdsensing tasks to each edge cloud with budget. The users who are interested in performing the tasks can submit their bids with the sensing data to the corresponding edge clouds for participating. Then, each edge cloud executes the truth discovery for each task. Meanwhile, the weight of each user is estimated in the truth discovery process. If necessary, the platform in the deep cloud can estimate the truth through the integrated value from multiple edge clouds. Finally, each edge cloud selects a subset of users as winners to maximize the quality of winners under the budget constraint, and determines the payment to winners. The whole process is illustrated by Figure 1.

We aim to present an Incentive Mechanism for Truth Discovery in Edge-assisted Large-scale Mobile Crowdsensing (IMTEC), which is a two stage incentive mechanism, consisting of the truth discovery stage and budget feasible reverse auction stage. In the truth discovery stage, IMTEC estimates the truth in the edge clouds and deep cloud (optional), and returns the weights of users. In the stage of budget feasible reverse auction, IMTEC selects the winners, and determines the payment to the users.

The problem of the designing incentive mechanism for the truth discovery in edge-assisted large-scale mobile crowdsensing is very challenging. First, different from the cloud based mobile crowdsensing systems, the edge-assisted large-scale mobile crowdsensing has two types of data processing resources: deep cloud and edge cloud. A new edge-cloud integrated truth discovery solution is needed to support either global or local truth discovery. Second, the estimated truth in different edge clouds may have different reliability due to the unbalanced user distribution over edge clouds. We should consider the reliability when we update the weight of edge cloud. Furthermore, the platform usually has a preference over the sensing tasks (e.g., priority according to the urgency). The objective function of our incentive mechanism should take into consideration both the preference over tasks and the contribution of users to truth discovery. Finally, the users may take a strategic behavior by submitting a dishonest bid price to maximize their utilities.

The main contributions of this paper are as follows:

- To the best of our knowledge, we are the first to present an integrated solution, which stimulates the strategic users to contribute for truth discovery in the edge computing based mobile crowdsensing.
- We present an edge-assisted large-scale mobile crowdsensing architecture, which enables the platform in the deep cloud to offload the sensing tasks to the edge clouds deployed in different geographical areas.
- We use the improved Conflict Resolution on Heterogeneous Data (CRH) [18,19] to estimate the truth for both of the deep cloud and edge cloud. Specifically, the truth discovery in deep cloud takes into consideration the reliability and the importance of estimated truth in edge clouds.
- We formulate the quality function based on the importance of tasks and the weight of users in truth discovery. We model the Budget Feasible Quality Optimization (BFQO) problem to maximize the quality function under the budget constraint. We show that the *BFQO* problem is a budget feasible submodular maximization problem, and design a budget feasible reverse auction mechanism to solve the BFQO problem based on a random mechanism and the proportional share allocation rule [20], which is computationally efficient, individually rational, truthful, budget feasible and a constant approximate.

The rest of the paper is organized as follows. Section 2 formulates the edge-assisted large-scale mobile crowdsensing model and the BFQO problem, and lists some desirable properties. Section 3 presents the detailed design of truth discovery. Section 4 presents the detailed design of budget feasible reverse auction. Performance evaluation is presented in Section 5. We review the state-of-art research in Section 6, and conclude this paper in Section 7.

## 2. System Model

In this section, we modeled the truth discovery in an edge-assisted large-scale mobile crowdsensing architecture as a reverse auction. Then we presented some desirable properties.

### 2.1. Edge-Assisted Mobile Large-scale Crowdsensing Model

As illustrated by Figure 1, we considered a mobile crowdsensing system consisting of a platform residing in the deep cloud, a set $E=\{e_{1},\;e_{2},\;\ldots ,\;e_{r}\}$ of *r* edge clouds, and a set U={1, 2, …, n} of *n* smartphone users, who are interested in performing tasks. The edge clouds are fixed local servers, such as base stations, access points, which can provide crowdsensing service for the users in the specific areas. Let $U_{k}$ be the set of users in the coverage area of edge cloud $e_{k}$, $U=\cup U_{k}$, k=1, 2, …, r. Without loss of generality, the coverage areas of edge clouds can have overlaps, i.e., any user can belong to multiple edge clouds.

The platform first distributes a set $T=\{t_{1},\;t_{2},\;\ldots ,\;t_{m}\}$ of *m* tasks to all edge clouds for large-scale mobile crowdsensing. Each edge cloud is with a budget, which can be determined by the importance of the sensing data in the coverage area of the edge cloud. Let $G=(G_{1},\;G_{2},\;\ldots ,\;G_{r})$ be the budget profile of all edge clouds. Each task $t_{j}\in T$ is associated with a type $\mu_{j}$, which represents the importance of task $t_{j}$. Let $\mu =(\mu_{1},\;\mu_{2},\;\ldots ,\;\mu_{m})$ be the types of all tasks.

Each edge cloud $e_{k}\in E$ records the tasks, and distributes them to the user subset $U_{k}$. Each user i∈U submits a triple $B_{i}=(T_{i},\;b_{i},\;X_{i})$ to the edge cloud it belongs to. $T_{i}$ is the task set he/she is willing to perform, and $b_{i}$ is his/her bid price that user *i* wants to charge for performing $T_{i}$. Each $T_{i}$ is associated with the cost $c_{i}$, which is the private information and known only to user *i*. Different from most crowdsensing systems [21,22,23,24], each user submits his/her sensing data $X_{i}$ of tasks in set $T_{i}$ with his/her bid price. Let $X=(X_{1},\;X_{2},\;\ldots ,\;X_{n})$ be the sensing data submitted by all users, where $X_{i}=(x_{i}^{1},x_{i}^{2},\ldots ,x_{i}^{\left|T_{i}\right|})$. Let $T^{k}$ and $X^{k}$ be the submitted tasks and the sensing data accordingly in the edge cloud $e_{k}$.

On receiving the sensing data, each edge cloud $e_{k}\in E$ computes the weight $w_{i}$ of any user $i\in U_{k}$ and estimates the truth $X_{*}^{k}$ for all sensing tasks based on the sensing data through truth discovery. Let $w^{k}$ be the weights of all users in $U_{k}$. Let $X_{*}$ be the estimated truth of all edge clouds.

Optionally, the edge clouds can submit the estimated truth to the deep cloud. The deep cloud computes the weight $g^{k}$ of any edge cloud $e_{k}\in E$ and estimates the truth $X_{**}$ for all sensing tasks based on $X_{*}$ through the truth discovery method. Note that the truth discovery in the deep cloud is an optional operation. It depends on the demand of the crowdsensing platform and/or the location dependence of the sensing data, etc. For example, the crowdsensing platform wants to collect the noise levels in different areas of an urban through the edge clouds in the areas. It can estimate the noise around a road through integration of noise levels from the edge clouds along the road. However, it is meaningless to integrate the noise levels of all edge clouds because the noise level is highly dependent on location.

Finally, each edge cloud conducts the budget feasible reverse auction. Given the task set $T^{k}$, user set $U_{k}$, budget $G_{k}$, task types μ and the bid profile $B^{k}=(B_{1},\;B_{2},\;\ldots ,\;B_{\left|U_{k}\right|})$, each edge cloud $e_{k}$ calculates the winner set $S_{k}\subseteq U_{k}$ and the payment $p_{i}$ for each winner $i\in S_{k}$. Let $S=\underset{k=1,2,\ldots ,r}{\cup }S_{k}$. Let $p^{k}$ and p be the payment profile of $S_{k}$ and S, respectively. 

We define the utility of any user *i* as the difference between the payment and its real cost:

(1) $$u_{i}=p_{i}-c_{i}$$

Specifically, we considered the cost of any loser could be ignored since the data quality is low. Thus the utility of any loser is zero.

Since we considered the users as selfish and rational individuals, each user could behave strategically by submitting a dishonest bid price to maximize its utility.

For any task $t_{j}\in T_{i}$, we defined the quality function obtained from the winner set $S_{k}$ in edge cloud $e_{k}$ as:

(2) $$V\left(S_{k}\right)=\sum_{t_{j}\in T^{k}}\mu_{j}\log\left(1+\sum_{i\in S_{k},t_{j}\in T_{i}}w_{i}\right),$$

where *log* term reflects the platform’s diminishing return on participating users.

The objective of the reverse auction in each edge cloud $e_{k}\in E$ is maximizing the quality function such that the total payment is no more than the budget. We referred to this problem as the Budget Feasible Quality Optimization (BFQO) problem, which can be formulated as follows:

(3) $$\mathrm{Objective}:\;\mathrm{Maximize}\;V\left(S_{k}\right)$$

(4) $$\mathrm{Subject}\;\mathrm{to}:\;\sum_{i\in S_{k}}p_{i}\leq G_{k}$$

### 2.2. Desirable Properties

Our objective was to design an incentive mechanism satisfying the following desirable properties:

- Computational efficiency: An incentive mechanism is computationally efficient if the truth, the winner set and the payment profile can be computed in polynomial time.
- Individual rationality: Each winner will have a non-negative utility while bidding its true cost, i.e., $u_{i}\geq 0,\;\forall i\in S.$
- Truthfulness: An incentive mechanism is truthful if reporting that the true cost is a weakly dominant strategy for all users. In other words, no user can improve its utility by submitting a false cost, no matter what others submit.
- Budget feasibility: In every edge cloud, the total payments to the winners are no more than the budget of the edge cloud, i.e., $\sum_{i\in S_{k}}p_{i}\leq G_{k}$, for $\forall e_{k}\in E$.
- Approximation: We attempted to find a solution with the highest possible value of quality function. For χ≥1, we said the incentive mechanism was the χ-approximate if the mechanism selects a winner set Π such that OPT≤χV(Π).

We listed the frequently used notations in Table 1.

## 3. Truth Discovery

### 3.1. Truth Discovery in Edge Clouds

We used the CRH truth discovery algorithm [18,19] to estimate the truth in edge clouds. There are two key steps in CRH: weight update and truth update.

(1) Weight Update

In this step, we assumed the estimated truth of each task was fixed. The basic idea is that a user’s weight should be assigned a high value if this user provides data, which is close to the estimated truth. For each edge cloud $e_{k}$, the weight of any user $i\in S_{k}$ is calculated as:

(5) $$w_{i}=f(\sum_{j\in T_{i}}d(x_{i}^{j},x_{*}^{j})),$$

where d(⋅) is a distance function which measures the difference between user observation values $x_{i}^{j}$ and the estimated truth $x_{*}^{j}$. f(⋅) is a monotonically decreasing function. In this paper, we adopted the logarithmic function as f(⋅) for any user $i\in S_{k}$ due to its good practical performance:

(6) $$f(\sum_{j\in T_{i}}d(x_{i}^{j},x_{*}^{j}))=\log\left(\frac{\sum_{i^{\prime }\in U_{k}}\sum_{j\in T_{i^{\prime }}}d(x_{i^{\prime }}^{j},x_{*}^{j})}{\sum_{j\in T_{i}}d(x_{i}^{j},x_{*}^{j})}\right)$$

The distance function d(⋅) depends on the application scenarios. For the sensing data, we could adopt the following normalized squared distance function straightforwardly:

(7) $$d\left(x_{i}^{j},x_{*}^{j}\right)=\frac{{\left(x_{i}^{j}-x_{*}^{j}\right)}^{2}}{std_{j}},$$

where $std_{j}$ is the standard deviation of all observation values for task $t_{j}$. 

(2) Truth Update

In this step, we assumed the weight of each user is fixed. Then we could estimate the truth of the task $t_{j}$ in edge cloud $e_{k}$:

(8) $$x_{*}^{j}=\frac{\sum_{i\in U_{k},j\in T_{i}}w_{i}x_{i}^{j}}{\sum_{i\in U_{k}}w_{i}}$$

The above two steps will be iteratively conducted until the estimated truth does not change or the iterations have been conducted φ times. The truth discovery in edge cloud is illustrated in Algorithm 1.
**Algorithm 1: Truth Discovery**
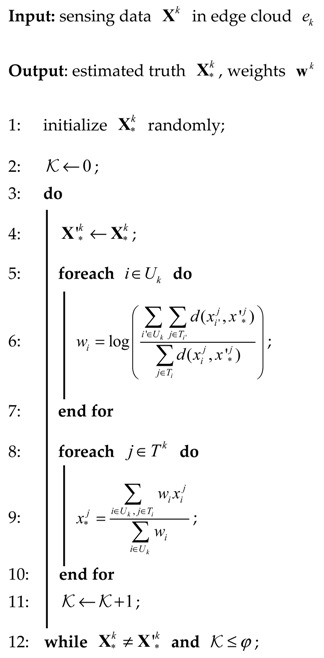


### 3.2. Truth Discovery in Deep Cloud

Basically, the truth discovery in the deep cloud is very similar to that in the edge cloud. The deep cloud estimates the truth based on the local truth of edge clouds. What is special is that we considered the reliability and the importance of estimated truth in edge clouds when calculating the weights of edge clouds. We updated the weight of any edge cloud $e_{k}$ as:

(9) $$g^{k}=\Delta^{k}\log\left(\frac{\sum_{k^{\prime }=1}^{r}\sum_{j\in T^{k^{\prime }}}d(x_{k^{\prime }}^{j},x_{*}^{j})}{\sum_{j\in T^{k}}d(x_{k}^{j},x_{*}^{j})}\right),$$

where $\Delta^{k}=(\alpha \frac{\left|U_{k}\right|}{\sum_{k^{\prime }=1}^{r}\left|U_{k^{\prime }}\right|}+\beta \frac{G_{k}}{\sum_{k^{\prime }=1}^{r}G_{k^{\prime }}})$,α, β are constants, α∈[0,1], β∈[0,1], α+β=1.

We considered that the reliability of the estimated truth of edge cloud depends on the number of users within it, and the importance of the estimated truth of edge cloud depends on the budget allocated in the edge cloud. 

## 4. Budget Feasible Reverse Auction

In this section, we proposed the budget feasible reverse auction, which was conducted in each edge cloud, to solve the BFQO problem defined in Formula (2) and Formula (3).

First, we gave the definition of submodular.

Definition 1 (Submodular Function). For a finite set Y, function $V:2^{Y}\to \mathbb{R}$ is submodular if

$$V\left(H_{1}\cup \{y\}\right)-V(H_{1})\geq V\left(H_{2}\cup \{y\}\right)-V(H_{2})$$

for any $H_{1}\subseteq H_{2}\subseteq Y$ and $y\in Y\backslash H_{2}$. Moreover, a submodular function V is non-decreasing if $V(H_{1})\leq V(H_{2})$ for any $H_{1}\subseteq H_{2}$.

Next, we showed that our quality function defined in Formula (2) was a non-negative non-decreasing submodular function.

**Theorem** **1.** *The quality function defined in Formula (2) is a non-negative non-decreasing submodular function.*

**Proof.**  According to Definition 1, we needed to show that $V\left(S_{k}\cup \{y\}\right)-V(S_{k})\geq V\left({S^{\prime }}_{k}\cup \{y\}\right)-V({S^{\prime }}_{k})$, for any $S_{k}\subseteq {S^{\prime }}_{k}\subseteq U_{k}$ and $y\in U_{k}\backslash {S^{\prime }}_{k}$. Considering $V\left(S_{k}\right)=\sum_{t_{j}\in T^{k}}\mu_{j}\log\left(1+\sum_{i\in S_{k},t_{j}\in T_{i}}w_{i}\right)$, we had

$$\begin{array}{ll} & V\left(S_{k}\cup \{y\}\right)-V(S_{k})\\ = & (\sum_{t_{j}\in T^{k}\backslash T_{y}}\mu_{j}\log(1+\sum_{i\in S_{k},t_{j}\in T_{i}\backslash T_{y}}w_{i})\;+\sum_{t_{j}\in T_{y}}\mu_{j}\log(1+\sum_{i\in S_{k}\cup \{y\},T_{i}\cap T_{y}\neq \emptyset ,t_{j}\in T_{y}}w_{i}))-(\sum_{t_{j}\in T^{k}\backslash T_{y}}\mu_{j}\log(1+\sum_{i\in S_{k},t_{j}\in T_{i}\backslash T_{y}}w_{i})\\ & +\sum_{t_{j}\in T^{k}\cap T_{y}}\mu_{j}\log(1+\sum_{i\in S_{k},t_{j}\in T_{i}\cap T_{y}}w_{i}))\\ = & \sum_{t_{j}\in T_{y}}\mu_{j}\log(1+\sum_{i\in S_{k}\cup \{y\},T_{i}\cap T_{y}\neq \emptyset ,t_{j}\in T_{y}}w_{i})-\sum_{t_{j}\in T^{k}\cap T_{y}}\mu_{j}\log(1+\sum_{i\in S_{k},t_{j}\in T_{i}\cap T_{y}}w_{i})\\ = & \sum_{t_{j}\in T^{k}\cap T_{y}}\mu_{j}\log(1+\sum_{i\in S_{k},t_{j}\in T_{i}\cap T_{y}}w_{i})\;+\sum_{t_{j}\in T_{y}\backslash T^{k}}\mu_{j}\log(1+w_{y})-\sum_{t_{j}\in T^{k}\cap T_{y}}\mu_{j}\log(1+\sum_{i\in S_{k},t_{j}\in T_{i}\cap T_{y}}w_{i})\\ = & \sum_{t_{j}\in T_{y}\backslash T^{k}}\mu_{j}\log(1+w_{y})\\ \geq & \sum_{t_{j}\in T_{y}\backslash {T^{\prime }}^{k}}\mu_{j}\log(1+w_{y})=V\left({S^{\prime }}_{k}\cup \{y\}\right)-V({S^{\prime }}_{k}) \end{array}$$

Therefore function V(⋅) is submodular. Obviously, our quality function was non-negative and non-decreasing. □

Since the quality function is a non-negative non-decreasing submodular function. The BFQO problem is a budget feasible submodular maximization problem actually. We applied the random mechanism proposed by Chen [25], which has been proved to achieve properties of individual rationality, budget feasibility, truthfulness and 1/5 approximation of the optimum [26], to select winners and determine the payment. Then, we selected all registered users with winning social neighbors as the agents, and allocated the reward based on the contribution to the value obtained by the platform. The whole process is illustrated in Algorithm 2.
**Algorithm 2: Budget Feasible Reverse Auction**
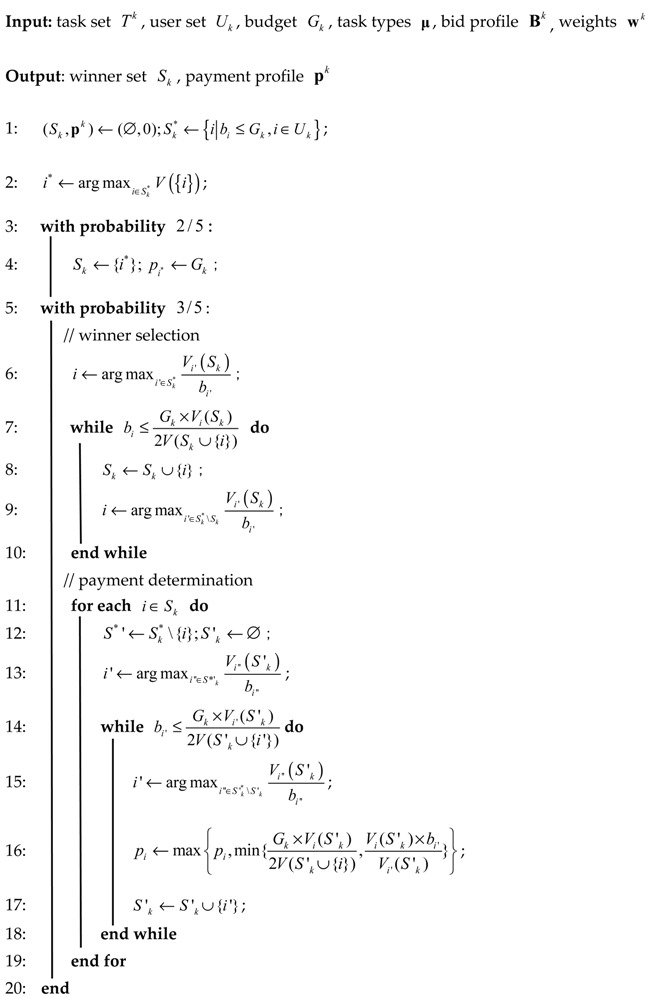


Let $S_{k}^{*}$ be the set of users whose bid price is no more than the budget. With probability $\frac{2}{5}$ (Lines 3–4), we selected the user $i^{*}$ with maximum quality in set $S_{k}^{*}$ as the winner, and the payment is equal to the budget. 

With probability $\frac{3}{5}$ (Lines 5–20), the reverse auction consists of the winner selection and payment determination phase. In the winner selection phase, we processed each user $i\in S_{k}^{*}\backslash S_{k}$ iteratively according its effective marginal quality $\frac{V_{i}(S_{k})}{b_{i}}$, where $V_{i}(S_{k})$ is the marginal quality over set $S_{k}$ of selected winners, i.e., $V_{i}(S_{k})=V(S_{k}\cup \{i\})-V(S_{k})$, (Lines 7–10). In each iteration, if the bid price is no more than $\frac{G_{k}\times V_{i}(S_{k})}{2V(S\cup \{i\})}$, the user i is included in the winner set. In the payment determination phase, for each winner $i\in S_{k}$, we executed the winner selection phase over $S_{k}^{*}\backslash \{i\}$, and denoted the winner set as ${S^{\prime }}_{k}$ (Lines 14–18). We applied the modified proportional share allocation rule [20] to achieve the critical value of payment. The payment for any winner i is

(10) $$p_{i}=\max_{i^{\prime }\in {S^{\prime }}_{k}}\left\{\min\{\frac{G_{k}\times V_{i}(S{{}^{\prime }}_{k}^{i^{\prime }-1})}{2V(S{{}^{\prime }}_{k}^{i^{\prime }-1}\cup \{i\})},\frac{V_{i}(S{{}^{\prime }}_{k}^{i{}^{\prime }-1})\times b_{i{}^{\prime }}}{V_{i^{\prime }}(S{{}^{\prime }}_{k}^{i^{\prime }-1})}\}\right\}$$

where $S{{}^{\prime }}_{k}^{i^{\prime }-1}$ is the winner set before we included $i^{\prime }$ into S′.

**Lemma** **1.** IMTEC is computationally efficient.

**Proof.**  We first analyzed the time complexity of truth discovery in each edge cloud (Algorithm 1). Updating the weight for all users in each edge cloud (lines 5–7) takes O(nm) time. Updating the truth for all tasks in each edge cloud (lines 8–10) takes O(nm) time. The number of iterations (lines 3–12) is at most φ. Thus the running time of Algorithm 1 is O(mn). The running time of truth discovery in deep cloud is O(mr) through the similar analysis of Algorithm 1. Since r<<n, the whole truth discovery takes O(mn) time.

Next, we analyzed the time complexity of budget feasible reverse auction (Algorithm 2). It suffices to analysis the time complexity of the second branch (Lines 5–20) of random mechanism since it dominates the running time of budget feasible reverse auction. Finding the user with maximum marginal density takes O(nm) time, where computing the value of $V_{i}(S_{k})$ takes O(m) time. Since there are m tasks and each winner should contribute at least one new task to be selected, the number of winners is at most m. Hence, the while-loop (Lines 7–10) thus takes $O(nm^{2})$ time. In each iteration of the for-loop (Lines 11–19), a process similar to Lines 7–10 is executed. Hence the payment determination takes $O(nm^{3})$. The running time of Algorithm 2 is dominated by the payment determination phase, which is bounded by $O(nm^{3})$. □

Note that the running time of Algorithm 2 is very conservative since the number of winners is much less than *n* in practice.

We could obtain the following theorem based on Lemma 1 and [26] straightforwardly.

**Theorem** **2.** IMTEC is computationally efficient, individually rational, truthful, budget feasible and 1/5 approximate.

## 5. Performance Evaluation

We conducted simulations to investigate the performance of IMTEC on the real experience data.

### 5.1. Simulation Setup

We first measured the performance of truth discovery, and compared it with the following two algorithms:

- Equal Reliability (ER): ER considers that each edge cloud is with the same reliability. This means that ER estimates the truth in the deep cloud through Formula (9) with α=0.
- Square Root Distance (SRD): SRD uses the distance function $d\left(x_{i}^{j},x_{*}^{j}\right)=\sqrt{|x_{i}^{j}-x_{*}^{j}|}$ instead of the normalized squared distance function given in Formula (7) to estimate the truth both in edge clouds and deep cloud.

To measure the precision of our truth discovery, we defined the MAPE (Mean Absolute Percentage Error) of the truth discovery as: $\sqrt{\frac{1}{m}\sum_{j=1}^{m}{\left|\frac{x_{*}^{j}-\tilde{x_{*}^{j}}}{\tilde{x_{*}^{j}}}\right|}^{2}}$, where $\tilde{x_{*}^{j}}$ is the ground truth of task $t_{j}$.

Then, we conducted the simulations to evaluate the budget feasible reverse auction, and compared it with the following benchmark algorithms.

- Approximate optimal: For any edge cloud $e_{k}\in E$, approximate optimal mechanism selects the winners from $U_{k}$ to maximize the quality with budget $G_{k}$. The problem is essentially a budgeted maximum coverage problem, which is a well-known NP-hard problem. It is known that the greedy algorithm provides (1−1/e) approximation solution [27]. Note that the approximate optimal mechanism is untruthful.
- Coverage function: The objective is maximizing the value function, defined as $V\left(S_{k}\right)=|\underset{i\in S_{k}}{\cup }T_{i}|$, such that the total payment is no more than the budget. In other words, the coverage function is the reverse auction, which aims to maximize the coverage of tasks.

For our simulations, we used the crowd temperature dataset [28], which was collected from taxi drivers of Rome using GPS to find directions. It included 1000 drivers, 32 tasks with ground truth and 5030 samples. Each sample contained the driver ID, driver’s location, the task ID and the outdoor temperature. We considered that the coverage area of each edge cloud was a circular area with radius of 3 km. We set up 16 edge clouds to cover the users’ geographic area. The platform sets μ according to the importance of specific tasks. In our experiment, we generated μ uniformly over [1,5]. The cost of each user was selected randomly from the auction dataset [29], which contained 5017 bid prices for Palm Pilot M515 PDA from eBay. We set the total budget as G=1000, and randomly assigned it to all edge clouds. The total number of users was 1000. In our truth discovery algorithm, we set the maximum number of iterations φ as 100 and initialized α=0.7,β=0.3. We would change these key parameters to verify the impact of them on the precision of truth discovery. Each measurement was averaged over 100 instances.

### 5.2. Evaluation of Truth Discovery

**Figure 2:**
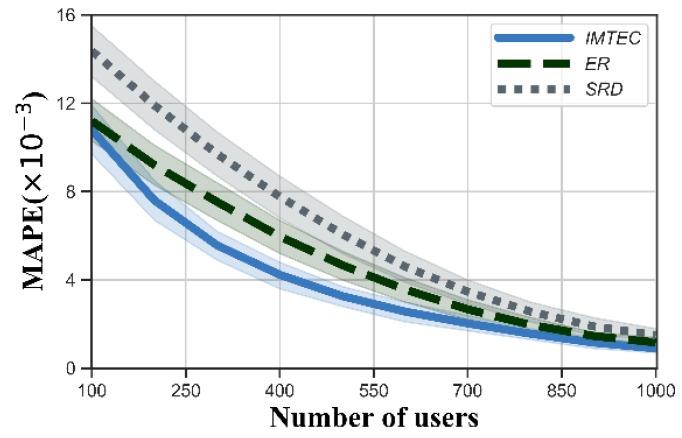
Mean Absolute Percentage Error (MAPE) versus the number of users.

First, we measured the precision of truth discovery for IMTEC, ER and SRD, and the MAPE is shown in Figure 2. We could see that the MAPE of all three algorithms gradually decreased with the increasing number of users. Among three algorithms, IMTEC could obtain the best precision. This is because IMTEC takes into account the number of users in the edge clouds so that the edge clouds with more users play an important role in truth discovery. Meanwhile, we could see that IMTEC used a more suitable distance function than SRD because IMTEC took account of the standard deviation of the submitted data of each task, and the tasks with large data deviations had less impact on updating users’ weights.

**Figure 3:**
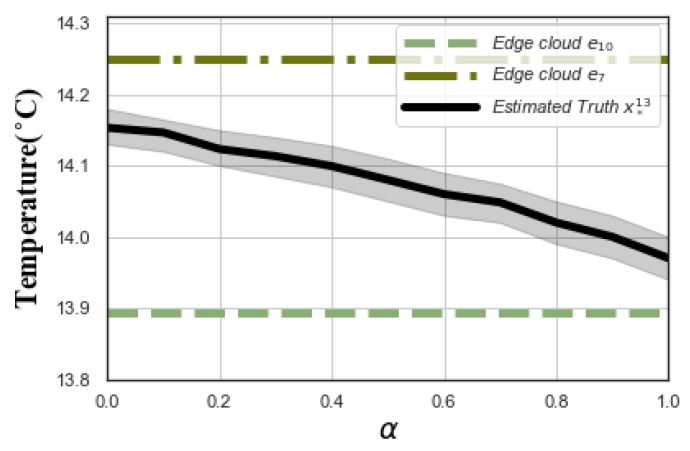
MAPE versus α ($\;G_{7}=300$,$G_{10}=100$).

Figure 3 depicts the impact of α and β on *MAPE*. Since α+β=1, we only changed the value of α. To reveal the impact, we selected two edge cloud, $e_{7}$ and $e_{10}$, with $|U_{7}|=104,\;G_{7}=300,\;|U_{10}|=245,\;G_{10}=100$, respectively. We set different α, and observed the estimated truth $x_{*}^{13}$ of task $t_{13}$ in the deep cloud. We could see that the estimated truth of deep cloud closed gradually to the estimated truth of edge cloud $e_{10}$. This is because when α increases, the deep cloud tends to assign high weight to the edge cloud with more users based on Formula (9).

**Figure 4:**
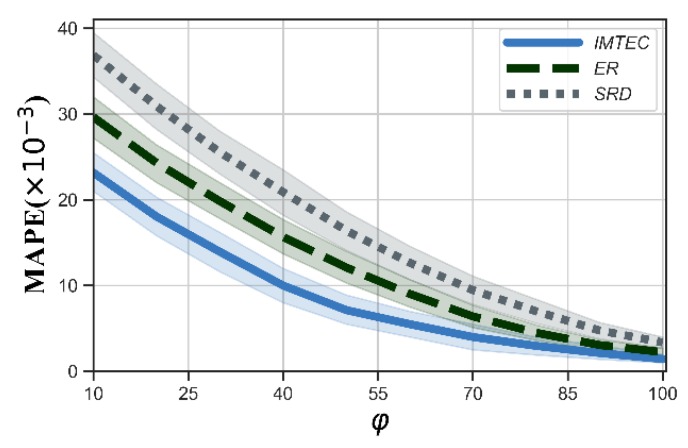
MAPE versus φ.

From Figure 4, we could see that the more the number of iterations φ is, the higher the precision will be for all three algorithms. Particularly, The MAPE of IMTEC was lower than 1% when φ≥40.

### 5.3. Evaluation of Reverse Auction

**Figure 5:**
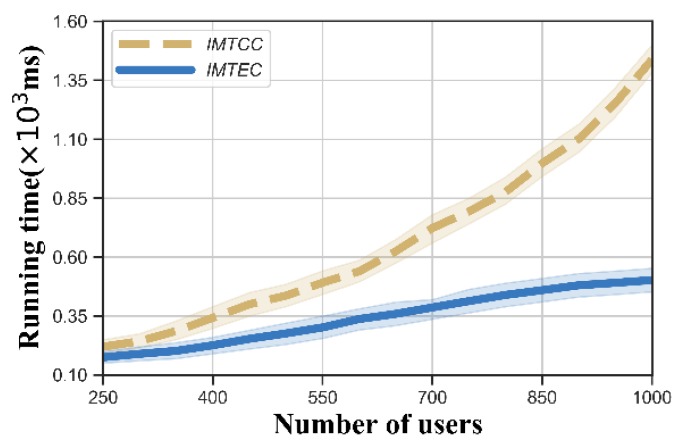
Running time versus number of users.

To evaluate the computational performance of IMTEC, we compared the total running time of IMTEC with the Incentive Mechanism for Truth Discovery in Cloud-assisted Large-scale Mobile Crowdsensing (IMTCC), which executes both the truth discovery stage and budget feasible reverse auction stage only in the deep cloud. We can see from Figure 5 that IMTEC shows great superiority in terms of running time. Even though there were 1000 users, our IMTEC could be terminated within 0.5 s, demonstrating great expandability. This is because the users of edge cloud are much fewer than those in the deep cloud, and the truth discovery stage in edge clouds can be performed in parallel.

**Figure 6:**
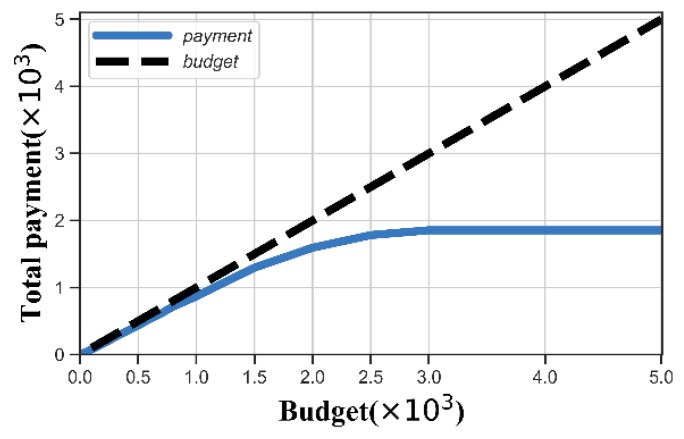
Payment versus budget.

We changed the budget of each edge cloud, and measured the average payment of each edge cloud. Figure 6 shows that the total payment is always no more than the budget. As the budget increased, the number of winners of each edge cloud also increased. Since the number of users in each edge cloud was fixed, the total payment to the winners became stable when the budget was large enough.

**Figure 7:**
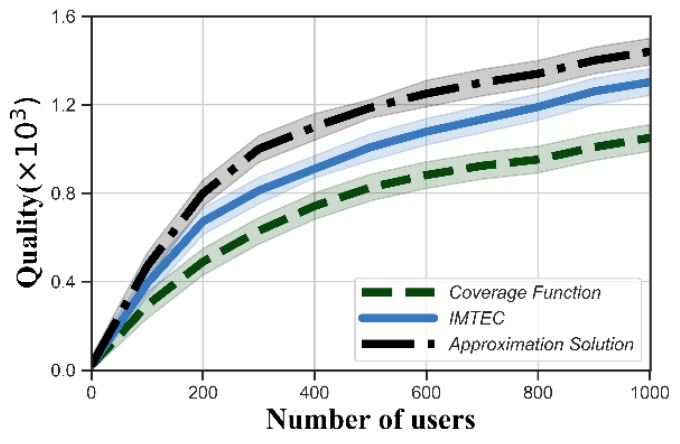
Quality function versus the number of users.

**Figure 8:**
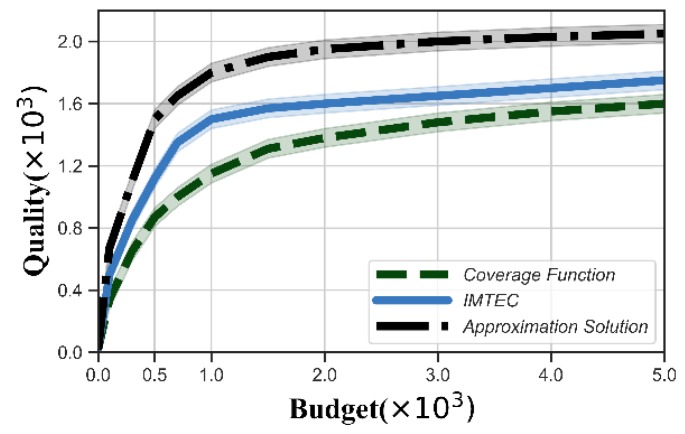
Quality function versus budget.

Figure 7 and Figure 8 show the values of the quality function obtained from IMTEC, approximation solution and coverage function. The quality increased with the number of users because the platform could select better users. The quality also increased with increasing budget, and became stable when the budget was large enough. We could see that our reverse auction improved the quality compared with the coverage function. This is because the coverage function aims to maximize the coverage of tasks rather than quality. Moreover, the quality obtained by IMTEC was very close to that obtained by the approximation solution.

**Figure 9:**
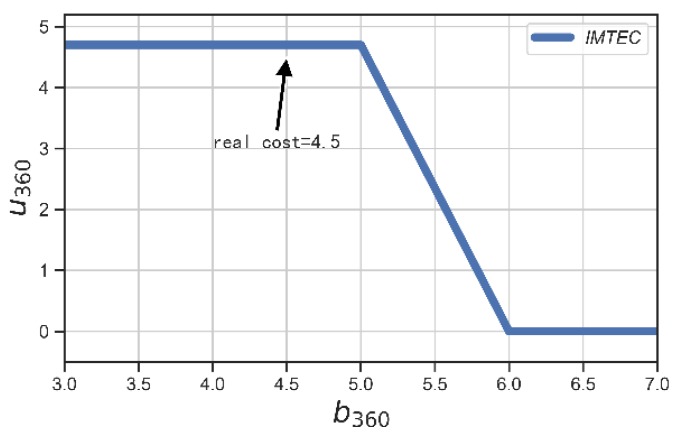
Utility of user with ID = 360 (winner).

**Figure 10:**
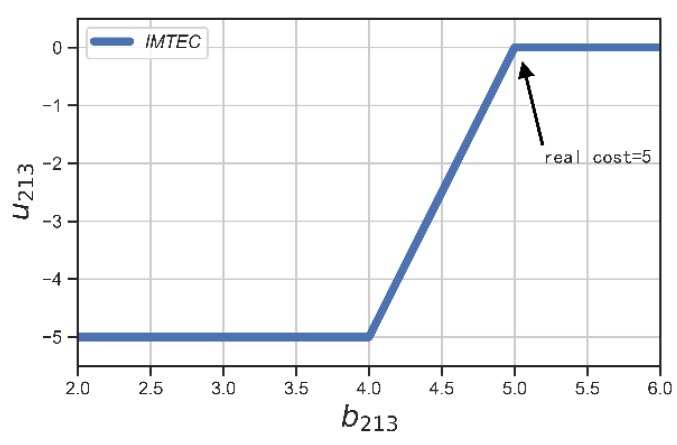
Utility of user with ID = 213 (loser).

We verified the truthfulness of IMTEC by randomly picking two users in our edge-assisted large-scale mobile crowdsensing system, and allowing them to bid prices that are different from their true costs. We illustrate the results in Figure 9 and Figure 10. We could see that the winner 360 achieved its maximum utility if he/she bids truthfully, i.e., $b_{360}=c_{360}=4.5$. Accordingly, the user 213 achieved its nonnegative utility if he/she bids truthfully, i.e., $b_{213}=c_{213}=5$.

### 5.4. Summary

From the simulations, we found that IMTEC could obtain high precision of truth estimation since it considers the reliability and the importance in edge clouds when calculating the weights of edge clouds. Comparing with the other distance function, the standard deviation adopted in IMTEC could reduce the impact of data deviations on updating users’ weights. Moreover, IMTEC could obtain high precision of estimated truth with a small number of iterations. Our simulations also verified that IMTEC outperformed cloud based mobile crowdsensing in terms of running time, and could obtain the sensing data with high quality, which was very close to the output of the untruthful approximate optimal mechanism.

## 6. Related Work

### 6.1. Mobile Corwdsensing with the Edge Computing Paradigm

Recently, some mobile crowdsensing systems based on the edge computing paradigm have been proposed. The conceptual design architecture of Robust Mobile Crowd Sensing (RMCS) and practical implementations are described in [7]. They also provided a case study of smart transportation to demonstrate the feasibility of the proposed RMCS framework. Wei et al. [8] proposed a fog-based privacy-preserving scheme to enhance the security of the vehicular crowdsensing network. The scheme is with the security properties, including non-deniability, mutual authentication, integrity and forward privacy. Ma et al. [9] proposed two privacy preserving reputation management schemes: Basic Privacy Preserving Reputation Management (B-PPRM) and Advanced Privacy Preserving Reputation Management (A-PPRM), for edge computing enhanced MCS to simultaneously preserve privacy and deal with malicious participants. Pu et al. [10] presented a novel hybrid edge computing framework integrated with the emerging edge cloud radio access network, called Chimera, and formulated a novel multivehicle and multitask offloading problem, aiming at minimizing the energy consumption of network-wide recruited vehicles serving heterogeneous crowdsensing applications, and meanwhile reconciling both application deadline and vehicle incentive. Yang et al. [11] presented a novel edge-mediated mobile crowd sensing system, namely EdgeSense, which works on top of a secured peer-to-peer network consisting of participants. Then they proposed a novel decentralized spatial-temporal crowdsensing framework based on parallelized stochastic gradient descent. However, there is no incentive mechanism designed for truth discovery to stimulate the strategic users to submit high quality data.

Some privacy-preserving incentive mechanisms are proposed for mobile crowdsensing. Wang et al. divided the life cycle of each crowdsensing task in MCS into four phases: task allocation, incentive, data collection and data publishing, and designed a privacy-preserving framework [30] for MCS to protect users’ privacy in the whole life cycle of MCS. Wang et al. proposed a personalized privacy-preserving task allocation framework [31] for mobile crowdsensing that can allocate tasks effectively while providing personalized location privacy protection. Each worker uploads the obfuscated distances and personal privacy budget to the server instead of uploading its true location or true distances to tasks. However, these studies depend on the cloud service, and cannot be applied to the edge computing based mobile crowdsensing systems.

### 6.2. Quality-aware Incentive Mechanims in Crowdsensing

Various quality-aware incentive mechanisms have been proposed for mobile crowdsensing systems. Jin et al. proposed INCEPTION [32], a system framework that integrates the incentive, data aggregation and data perturbation. Wang et al. studied the problem of measuring users’ long-term quality, and they propose MELODY [33]. Wen et al. proposed an incentive mechanism based on a quality driven auction [34], where the user is paid off based on the quality of sensed data instead of the working time. Jin et al. designed an incentive mechanisms based on reverse combinatorial auctions, and incorporated the Quality of Information (QoI) of users into the incentive mechanism [35]. Xu et al. proposed a lightweight, multi-metric comprehensive, and parameter-free user quality evaluation method in the social mobile crowdsensing architecture, and used a reverse auction to optimize the new criterion, which takes both social cost and user quality into consideration [36]. Li et al. designed two quality-aware contract-based incentive mechanisms for crowdsensing, named QUAC-F and QUAC-I, under the full information model and incomplete information model, respectively [37]. Jin et al. proposed a payment mechanism, named Theseus [38], to incentivize high-effort sensing from workers. Theseus ensures that, at the Bayesian Nash Equilibrium of the non-cooperative game, all participating workers will spend their maximum possible effort on sensing. Zhang et al. analyzed the data integrity of mobile crowdsensed data in a user study of 60 people who participate in a crowdsensing campaign that collects barometric pressure data from various locations of campus [39]. Nava et al. presented a framework [40] for MCS that includes a model to represent the behavior of the users and a novel incentive mechanism. The model aims to characterize the behavior of users considering the availability of their resources and the non-homogeneity of their responses. A novel method to evaluate the trustworthiness of data contributed by users that also considers the subjectivity in the contributed data was proposed in [41]. The method is based on a comparison of users’ trust attitudes and applies nonparametric statistic methods. Yang et al. designed an unsupervised learning approach [42] to quantify the users’ data qualities and long-term reputations, and exploit an outlier detection technique to filter out anomalous data items. Furthermore, they proposed a Shapley value-based method to determine each user’s payment. However, none of these studies can be applied to the mobile crowdsensing based on the edge computing paradigm.

Overall, there is no off-the-shelf incentive mechanism designed in the literature for the mobile crowdsensing system to stimulate the strategic users to contribute for truth discovery in the edge-assisted mobile crowdsensing.

## 7. Conclusions

In this paper, we presented an edge-assisted large-scale mobile crowdsensing architecture, and developed an integrated solution for stimulating users to improve the quality of sensing data in this new architecture. We formulated the quality function and modeled the BFQO problem to maximize the quality function under the budget constraint. Further, we presented an incentive mechanism consisting of the truth discovery stage and budget feasible reverse auction stage. The first stage estimates the truth for both of deep cloud and edge cloud. In the second stage, we showed that the BFQO problem is a budget feasible submodular maximization problem, and designed a budget feasible reverse auction mechanism to solve the BFQO problem. We demonstrated that the proposed incentive mechanisms achieved computational efficiency, individual rationality, truthfulness, budget feasibility and constant approximation. The simulations show that our incentive mechanism was much faster than the traditional cloud based incentive mechanism, and can output more precise truth than the benchmark algorithms.

## Figures and Tables

**Table 1:** Frequently used notations.

Notation	Description
E,$e_{k}$	edge cloud set, edge cloud *k*
$T,\;t_{j}$	task set, task *j*
$T_{i},\;T^{k}$	task set of user *i*, task set of edge cloud *k*
$\mu ,\;\mu_{j}$	type set of tasks, type of task *j*
$G,\;G_{k}$	budget profile, budget of edge cloud *k*
m, n, r	number of tasks, number of users, number of edge clouds
$U,\;U_{k}$	user set, user set of edge cloud *k*
$S,\;S_{k}$	winner set, winner set of edge cloud *k*
$B^{k},B_{i}$	bid profile of users in edge cloud k, bid of user *i*
$b_{i},\;c_{i}$	bid price of user *i*, cost of user *i*
$X,\;X^{k}$	all sensing data, sensing data of edge cloud *k*
$X_{i},\;x_{i}^{j},\;x_{*}^{j}$	sensing data of user *i*, sensing data of user *i* for task *j*, estimated truth of task *j*
$X_{*}^{k},\;X_{*},\;X_{**}$	estimated truth of edge cloud *k*, estimated truth of all edge clouds, estimated truth of deep cloud
$w^{k},\;w_{i},\;g^{k}$	weights of all users in $U_{k}$, weight of user *i*, weight of edge cloud *k*
$p^{k},\;p,\;p_{i}$	payment profile of $S_{k}$, payment profile of S, payment to user *i*
$u_{i}$	utility of user *i*
$V\left(\cdot \right),\;V_{i}(\cdot )$	quality function, marginal quality of user *i*
d(⋅)	distance function
φ	maximum number of iterations for truth discovery
α, β	parameters of reliability and importance for truth discovery in deep cloud
